# Perception of University Students Towards National Efforts at Controlling COVID-19 Pandemics, the Practice of Prevention Measures and its Associated Factors During School Reopening

**DOI:** 10.3389/fpubh.2022.843974

**Published:** 2022-04-27

**Authors:** Mesfin Tadese, Saba Desta Tessema, Girma Altaye, Getaneh Baye Mulu

**Affiliations:** ^1^Department of Midwifery, College of Health Sciences, Debre Berhan University, Debre Berhan, Ethiopia; ^2^Department of Biology, College of Natural and Computational Sciences, Hawassa University, Hawassa, Ethiopia; ^3^Department of Nursing, College of Health Sciences, Debre Berhan University, Debre Berhan, Ethiopia

**Keywords:** COVID-19, perception, practice, factors, Ethiopia

## Abstract

**Background:**

The Coronavirus disease 2019 (COVID-19) pandemic has affected many communities including students. Even if restrictions are being lifted in some countries, i.e., Ethiopia, COVID-19 is not gone yet. Adjusting to the “new normal”, an emerging prevention response to the virus, can greatly recover public health and education. Thus, this study aimed to assess students' perception of national efforts at controlling the COVID-19 pandemic, including approaches to prevention measures, and associated factors during campus re-entry.

**Methods and Materials:**

We conducted a cross-sectional study among 682 Debre Berhan University (DBU) students from December 1 to 15, 2020, when students had just gone back to school. The data was entered into Epi-Data version 4.6 and exported to SPSS version 25.0 statistical software for analysis. The perception and practice of the participants were assessed using a scoring system. Binary logistic regression was run to identify the significant (*p* ≤ 0.05) predictors of COVID-19 prevention practice.

**Result:**

The overall high perception and good practice of prevention behaviors were 32%, 95% CI (28.8–35.2), and 37.5%, 95% CI (33.7–41.2), respectively. Being female [AOR (CI) = 1.67 (1.17–2.37)], have a rural residence [AOR (CI) = 1.56 (1.07–2.29)], fathers' education [AOR (CI) = 1.94 (1.06–3.56)], having respiratory disease [AOR (CI) = 2.81 (1.32–5.95)], and information sources from YouTube [AOR (CI) = 1.87 (1.19–2.91)] were significant factors for COVID-19 prevention practice. Besides, a high perception of national efforts at controlling COVID-19 [AOR (CI) = 2.94 (2.04–4.25)] was positively associated with the practice of prevention measures.

**Conclusion:**

During school reopening, most students had a low perception of national efforts at controlling COVID-19 and poor prevention practices. Socio-demographics, having a chronic illness, information sources, and perception of national efforts were factors of COVID-19 prevention practice. Thus, raising the perception of the national efforts, promoting precautionary measures, managing chronic illnesses, and disseminating information through YouTube are critical to preventing and controlling COVID-19 during campus re-entry.

## Introduction

Throughout history, human beings have had experiences fighting disastrous diseases. In some instances, the diseases are controlled and in others they continue and become an epidemic (1). In Wuhan, China, a novel COVID-19 outbreak was first reported in January 2020 (2). The World health organization (WHO) declared it a pandemic on March 11, 2020 (3). COVID-19 is an infectious viral pandemic disease caused by a new strain of an RNA virus called Severe Acute Respiratory Syndrome Coronavirus 2 (SARS-CoV2) (4). The virus is primarily transmitted through respiratory droplets and close contact from human to human. Poor hand hygiene practice, overcrowding, and close physical contact like handshaking can aggravate the spread of infection (5, 6). Fever, cough, myalgia, or fatigue are common and other symptoms like headache, sputum production, hemoptysis, and diarrhea are less common symptoms of COVID-19 (7).

The COVID-19 pandemic has affected almost all continents of the world, with over 117,455,738 confirmed cases and more than 2,605,472 deaths reported until March 8, 2021. America and Asian countries were highly affected. In Ethiopia, since the first case was detected on March 13, 2020, 165,029 confirmed cases and 2,420 deaths were reported up to March 8, 2021 (8). Compared to developed countries, the number of cases and deaths was low in Africa. This could relate to poor testing capacity, weak contact tracing, and poor reporting systems (9).

The practice of COVID-19 prevention measures was 87.94% in China (10), 56.4% in Iran (11), 36.5% in Pakistan (12), and 65% in South Wollo (13). Older age, urban residence, being of female gender, having a higher income level, greater risk perception, high self-efficacy, favorable attitude, and good knowledge of COVID-19 were some of the reported factors associated with COVID-19 prevention practice (10, 13–15). In addition, low perception of the disease and lack of trust in the prevention measures were major barriers to practicing COVID-19 prevention measures (16).

Across the world, in many countries, a national state of emergency and preventive measures were implemented. These include contact tracing, strict quarantine, social distancing, and universal use of masks, which are implemented to cut off the transmission and control the pandemic (17, 18). The Ethiopian government also implemented various synergistic strategies from closing all schools to declaring a state of emergency (19). Yet, the world is at risk of facing a recurrent epidemic. However, adapting to the “COVID-19: the new normal” prevention response, the Ethiopian Ministry of Health planned to reopen previously restricted education. Currently, students are returning to campuses with strict adherence to national preventive measures (20). The term “new-normal” describes an adjustment process or working side by side while preventing the COVID-19 pandemic. Recommendations advised people to stay safe and avoid the three C's: confined and closed spaces, crowded places, and close-contact settings whether at work, home, or school (21).

Debre Berhan University has enumerated several strategies to alleviate the spread of COVID-19 during the teaching-learning process. A physical distance should be maintained of 1–1.5 m in the library, laboratories, or workshops, while the distance in the classroom should be 1.5–2 m. Students must not participate in major sporting events. Classroom sizes should not exceed 30 and be limited to 40-min sessions. All staff and students should wear face masks at all times in public spaces and are required to frequently clean their hands using soap and water or using hand sanitizer. In addition, classrooms, offices, libraries, and laboratory rooms are ventilated and cleaned regularly at the end of each session. Those who develop COVID-19 symptoms should stay in their dorms and immediately report it and seek medical care.

Many studies have shown that the success of national efforts at controlling the pandemic profoundly depends on the community's perception and attitude toward the epidemic and the significance of prevention behaviors (22, 23). The perception and practice of students are strongly correlated with incidences of many infectious diseases, which negatively or positively affect efforts to prevent infectious transmission (22, 23). As the existence of a single case can cause pandemics, special consideration should be given to high-risk groups (20). Thus, assessing the practice and perception of COVID-19 preventive measures plays a vital role in understanding the sustainability of these behaviors. Therefore, this study aimed to assess the perception toward national efforts at controlling COVID-19 pandemics and the practice of prevention behaviors and its associated factors among Debre Berhan University students.

## Methods and Materials

### Study Area, Period, and Design

An institution-based cross-sectional study was undertaken at Debre Berhan University from December 1 to 15, 2020, when students re-entered campus. Debre Birhan is the capital city of the North Shewa Zone of the Amhara Region and is located 130 km Northeast of Addis Ababa on the Ethiopian highway. DBU has fourteen colleges and 50 departments. During the study year, DBU had 11,573 regular students, including 7,154 male students and 4,419 female students.

### Sample Size Determination and Sampling Procedure

Open Epi version 3.03 statistical software was used to calculate the required sample size. The assumptions are a practice of prevention measure of 65% (13), confidence level of 95%, a significance level of α = 5%, a margin of error of 5%, power (1-β) of 80%, and design effect was 2. Adding a 5% non-response rate, the sample size was 713.

A multistage sampling method was applied to recruit study participants. In the beginning, five colleges were randomly selected by the lottery method. Using probability proportional to size (PPS), the required sample size was allocated to the study departments depending on the number of students. Finally, a simple random sampling technique was followed to pick the required participants from the list of students obtained from the study departments.

### Inclusion and Exclusion Criteria

Randomly selected students who agreed to participate were included. Students who had a hearing problem and were unable to fill out the questionnaire were excluded from the study.

### Data Collection Tool and Measurements

Data were collected using a semi-structured, pre-tested, and self-administered questionnaire. The questionnaire was designed by referring to former studies (24–27) and was modified to fit the local setting. The questionnaire comprised questions related to sociodemographic background, information sources, the practice of COVID-19 prevention measures, and perceptions of the national effort at controlling the pandemic.

To determine the perception of students toward the national effort to control COVID-19, four questions with three alternative options, “Yes”, “Not sure”, or “No” were prepared (**Table 2**). For those who respond “Yes”, one point was allocated and those who responded “Not sure” or “No” received zero points. Students who scored below 3 points were considered to have low perception and those who scored 3 and above were considered to have a high perception of the national effort of COVID-19 prevention strategies (28). The reliability of items has been checked (Cronbach's alpha 0.66).

The preventive practice of COVID-19 was assessed by ten questions with three alternative responses “Always”, “Sometimes”, or “Never” (**Table 3**). Two points were given for the correct answers, one point for the answer “Sometimes”, and zero for incorrect answers. The total preventive practice scores were computed out of 20 points, and those who scored ≥75% (≥15 points) were considered to have good practices and <75% (<15 points) poor practices (27). The reliability of items has been checked (Cronbach's alpha 0.80).

### Data Quality Control

A pre-test was done among Victory College students on 5% (36 students) of the sample. A reproductive health expert checked the face and content validity of the questionnaire. The completeness, clarity, and appropriateness of questions were revised based on the results of the pretest and expert opinion. Two supervisors and four data collectors were involved in the data collection. One-day training regarding the objectives, procedures, and data collection tools were given to supervisors and data collectors. The supervisor and principal investigators checked and reviewed the data daily for completeness, clarity, and consistency.

### Data Management and Analysis

The data were systematically checked, cleaned, and coded. Epi-data version 4.6 was used for data entry and SPSS version 25.0 for statistical analysis. Basic descriptive analysis was performed. The means and standard deviations were estimated for continuous variables, and the frequency distribution was used for categorical data. Binary logistic regression analysis was computed to identify significant associates of COVID-19 prevention practice. A *p*-value of less than 0.05 in the multivariable analysis was considered statically significant. The result of this model was expressed using an adjusted odds ratio with 95% CI and the Hosmer Lemeshow goodness of fit was run to check for model fitness.

### Ethical Consideration

The Institutional Review Board (IRB) of Debre Berhan University approved the proposal, tools, and informed consent. After explaining the objective and benefits of the study, written informed consent was obtained from voluntary participants. Confidentiality and anonymity were ensured.

## Results

### Baseline Characteristics of Participants

Of the total 712 students, 682 (95.8% response rate) responded to the self-administered survey. The mean age of respondents was 23.35 ± 3.46 (SD) years. Up to 43.4% were females compared to 56.6% males. Besides, 76.2% were single, 91.8% were Christian and 83.6% were undergraduate students. Telegram (66.6%) was the commonest means of acquiring information regarding COVID-19. Around 4.7% and 4.4% of students had diabetes and hypertension, respectively (Table 1).

**Table 1:** Respondents baseline characteristics, 2020.

**Variables**	**Category**	**Frequency**	**Percent (%)**
Age	Mean ± SD of age (in years)	23.35 (mean)	± 3.46 (SD)
Gender	Male Female	386 296	56.6 43.4
Residence	Rural Urban	350 332	51.3 48.7
Religion	Christian Muslim	626 56	91.8 8.2
Marital status	Single In relationship Married Divorced/Widowed	520 76 72 14	76.2 11.1 10.6 2.1
Level of education	Undergraduate studies Postgraduate studies	570 112	83.6 16.4
Faculty	Medicine and Health Science Non-health/non-medical	442 240	64.8 35.2
Fathers education	No formal education Primary Secondary Higher education	304 112 104 162	44.6 16.4 15.2 23.8
Mothers education	No formal education Primary Secondary Higher education	360 112 100 110	52.8 16.4 14.7 16.1
Number of students in the dorm	<4 ≥4	112 560	17.9 82.1
Chronic disease	Diabetes Hypertension Kidney disease Heart disease Respiratory disease HIV/AIDS	32 30 26 40 40 24	4.7 4.4 3.8 5.9 5.9 3.5
Sources of information	Facebook Telegram You tube WhatsApp Instagram Scientific websites and articles TV and/or Radio Magazines and newspaper	376 454 152 50 86 84 408 78	55.1 66.6 22.3 7.3 12.6 12.3 59.8 11.4

### Perception of National Efforts at Controlling the COVID-19 Pandemic

In this study, 32% (*N* = 218), 95% CI (28.8–35.2), of students had a high perception of the national efforts at controlling the COVID-19 pandemic. The mean cumulative score of perception was 1.88 ± 1.4. More than half of the respondents (57.2%) agreed that the Ethiopian government had taken preventative measures early. However, only 42.5% think health facilities have adequate instruments to handle confirmed and suspected cases (Table 2).

**Table 2:** Perception of national efforts at controlling the COVID-19 pandemic among university students, 2020.

**Variables**	**Yes, *n* (%)**	**Not sure, *n* (%)**	**No, *n* (%)**
Do you believe that the Ethiopian government took preventative measures early?	390 (57.2)	196 (28.7)	96 (14.1)
Do you believe that the Ethiopian government took sufficient measures during the pandemic?	292 (42.8)	244 (35.8)	146 (21.4)
Do you believe that health facilities have adequate instruments to handle confirmed and suspected cases?	290 (42.5)	212 (31.1)	180 (26.4)
Do you believe that the efforts at your place of residence will succeed in restricting the spread of the virus?	312 (45.7)	256 (37.5)	114 (16.7)

### The Practice of COVID-19 Prevention Behaviors

Nearly three-quarters (72.4%) of participants regularly use face masks and 61% of them frequently washed their hands with soap and water. Besides, 60.7% of students self-quarantine if exposed and advised to do so. However, only 30.8% always use hand sanitizers. The overall good practice of prevention behaviors was 37.5% (*N* = 256), 95% CI (33.7–41.2), and the mean cumulative score of practice was 13.4 ± 4.04 (Table 3).

**Table 3:** COVID-19 prevention behavior practices among university students, 2020.

**Variables**	**Always**	**Sometimes**	**Never**
I wear a face mask.	494 (72.4%)	178 (26.1%)	10 (1.5%)
I avoid touching my nose, face, and eye with an unclean hand.	296 (43.4%)	310 (45.5%)	76 (11.1%)
I avoid handshakes and kissing others.	230 (33.7%)	324 (47.5%)	128 (18.8%)
I maintain physical distancing.	230 (33.7%)	332 (48.7%)	120 (17.6%)
I use soap and water to frequently clean my hands.	416 (61.0%)	242 (35.5%)	24 (3.5%)
I use hand sanitizers.	210 (30.8%)	278 (40.8%)	194 (28.4%)
I keep rooms well-ventilated.	268 (39.3%)	284 (41.6%)	130 (19.1%)
I avoid crowds.	266 (39.0%)	316 (46.3%)	100 (14.7%)
I use my bent elbow or tissue while coughing.	352 (51.6%)	262 (38.4%)	68 (10.0%)
I quarantine myself if am suspected to have COVID-19.	414 (60.7%)	154 (22.6%)	114 (16.7%)

### Factors of COVID-19 Prevention Practice

Based on the multivariable logistic regression analysis model, female students were 67% more likely to have good COVID-19 prevention practices compared to males [AOR (CI) = 1.67 (1.17–2.37)]. Students from rural residences were also 56% more likely to practice COVID-19 prevention measures compared to their counterparts [AOR (CI) = 1.56 (1.07–2.29)]. There are 94% increased odds of COVID-19 prevention practice among students whose fathers had attended higher education [AOR (CI) = 1.94 (1.06–3.56)] compared to those students whose fathers had no formal education. Similarly, the odds of having good practice of COVID-19 prevention behaviors were approximately three times higher among students who had the respiratory disease [AOR (CI) = 2.81 (1.32–5.95)] and high perception of the national efforts at controlling COVID-19 infection [AOR (CI) = 2.94 (2.04–4.25)]. Besides, students who obtained information from YouTube were 87% more likely to show COVID-19 prevention behaviors [AOR (CI) = 1.87 (1.19–2.91)] (Table 4).

**Table 4:** Factors of COVID-19 prevention behaviors, 2020.

**Variables**	**Categories**	**Practice**		**COR (95% CI)**	**AOR (95% CI)**
		**Unsatisfactory**	**Satisfactory**		
Gender	Male Female	260 166	126 130	1 1.62 (1.18–2.21)	1 1.67 (1.17–2.37)*
Residence	Rural Urban	210 216	140 116	1.24 (0.91–1.69) 1	1.56 (1.07–2.29)* 1
Religion	Christian Muslim	384 42	242 14	1.89 (1.01–3.53) 1	1.71 (0.88–3.31) 1
Faculty	Health Non-health	264 162	178 78	1.40 (1.01–1.95) 1	1.22 (0.83–1.79) 1
Fathers education	No formal education Primary Secondary Higher education	194 68 70 94	110 44 34 68	1 1.14 (0.73–1.78) 0.86 (0.53–1.37) 1.28 (0.86–1.88)	1 1.42 (0.83–2.45) 1.31 (0.69–2.46) 1.94 (1.06–3.56)*
Friend/family history of COVID-19	Yes No	54 372	44 212	1.43 (0.93–2.20) 1	1.67 (0.98–2.83) 1
Friends/families died from COVID-19	Yes No	20 406	22 234	1.91 (1.02–3.57) 1	0.80 (0.37–1.76) 1
Respiratory disease	Yes No	20 406	20 236	1.72 (0.91–3.26) 1	2.81 (1.32–5.95)* 1
Kidney disease	Yes No	20 406	6 250	0.49 (0.19–1.23) 1	0.35 (0.12–1.03) 1
Telegram use	Yes No	276 150	178 78	1.24 (0.89–1.73) 1	1.44 (0.98–2.10) 1
You tube	Yes No	88 338	64 192	1.28 (0.89–1.85) 1	1.87 (1.19–2.91)* 1
Magazines/Newspaper	Yes No	56 370	22 234	0.62 (0.37–1.04) 1	0.56 (0.31–1.04) 1
Websites/Articles	Yes No	62 364	22 234	0.55 (0.33–0.92) 1	0.56 (0.30–1.05) 1
Perception toward the national effort	Low perception High perception	326 100	138 118	1 2.79 (1.99–3.88)	1 2.94 (2.04–4.25)*

Bold and * indicate statistically significant at p < 0.05.

## Discussion

This study determined the perception and practice of university students regarding COVID-19 and its related factors to infection prevention practices. The levels of good practice toward COVID-19 were 37.5%, 95% CI (33.7–41.2).

The overall prevention practice was consistent with study findings from Pakistan (12), Mizan Tepi (20), Gondar (29), and Dire Dawa (30), in which 36.5, 42, 41.6, and 40.7% of university students had good prevention practices, respectively. This low practice might be the cause of the high number of infected cases and deaths in Ethiopia. Besides, the communities are careless and ignorant of the impacts of the pandemics. The people undermine the virus thinking that they are not vulnerable/infected and the virus is self-limiting. Others believed that the government was using it for political benefit rather than to combat the pandemic (30).

On the other hand, it was lower than 60.8% from Cameroon (31), 65% from South Wollo (13), and 59.8% from Addis Ababa (32). This could be related to the difference in the cut-values, i.e., this study used a 75% cut off point to categorize prevention practice while former studies used 80% and above. Besides, it may be due to variations in the sample size and study period. Our study was conducted during school resumption when students had just gone back to school, however, the Cameroon, South Wollo, and Addis Ababa studies were conducted during the rapid spread of the virus, April to May 2020. This was the time that most communities perceive the virus as dangerous and practice precautionary actions.

This level was, however, higher than the findings from South Ethiopia (33), in which only 20% of participants practiced COVID-19 prevention behaviors. The differences might have been subjected to variations in the study populations. This study sampled university students, who were supposed to have a better educational background and access to information. By contrast, the study in South Ethiopia included community members, where most are rural residents and attended primary school.

This study found that about one-third (32%) of students had a high perception of national efforts to control the COVID-19 pandemic. About 57.2% of students agreed that the Ethiopian government had taken preventative measures early. Only 42.5% think that health facilities have adequate instruments to handle confirmed and suspected cases. This was comparable with the study report from Egypt and Nigeria, where only 22% of populations are happy with their country's approach to the pandemic. However, 62.1% had a satisfactory perception of worldwide attempts at controlling and preventing the spread of COVID-19 (25). Moreover, in Libya, 73.8% of students are confident that their country can win the battle against COVID-19 (27).

Female students were more likely to have good COVID-19 prevention practices compared to male students [AOR (CI) = 1.67 (1.17–2.37)]. Similarly in Hong Kong male participants were less likely to implement prevention measures (15). Another study in Ethiopia found that female students had better compliance with prevention measures (34). Many studies concluded that female students are more cautious, sensitive, and preventive (24, 30). This might be because most female students spend their time at home, and are commonly involved in childcare and food preparation. Thus, by nature they are likely to exercise precautionary measures, i.e., cleaning washing, and avoiding crowds, to protect themselves and others. Besides, female participants in this study had a high-risk perception of COVID-19 (35). Perception of greater risk is associated with the adoption of health preventive behaviors (36).

Students from rural residences were more likely to practice COVID-19 prevention measures compared to their counterparts [AOR (CI) = 1.56 (1.07–2.29)]. Comparable findings were reported from Mizan Tepi University, where rural residency was positively associated with practice (20). This contradicts a study from China (37), Gondar (29), and South Wollo (13), where the odds of practice among students from urban residences were more likely than those from rural communities. The current study implies that information dissemination through social and news media, mobile phones, and public information campaigns has not substantially influenced the use of prevention measures, as it is anticipated that living in urban areas helps people access updated information. Further research may be needed to clarify these discordant findings.

Prevention practice was significantly higher among students whose fathers had attended higher education compared to students whose fathers had not attended formal education [AOR (CI) = 1.94 (1.06–3.56)]. Similarly, in southern Ethiopia, a significant positive association was reported (33) Furthermore, an online survey indicated poor prevention practices among people with a lower level of education (34). This could be because educated people are more likely to use social media, scientific websites, articles, and news media, which in turn increases access to information regarding precaution measures. Besides, the educational level was significantly related to knowledge of COVID-19 prevention behaviors (38). Consecutively, good knowledge increases the chance of adopting health prevention practices (29).

Increased odds of practice were noted among students with respiratory disease. Students who had a chronic respiratory disease were approximately three times to have good prevention practice [AOR (CI) = 2.81 (1.32–5.95)]. Similarly, in Dire Dawa (30) and Gedeo Zone (33), participants with chronic medical illnesses, i.e., diabetes, hypertension, chronic respiratory disease, and heart disease were more likely to practice prevention methods. The justification might be that patients with chronic illness have altered local/systemic immune responses and host microbiome. Thus, patients with systemic illness had a higher risk of developing severe disease from the virus, hospital admission, and death. In one meta-analysis study, patients with chronic obstructive pulmonary disease (COPD) had five times more risk of severe infection (39). Hence, they have a high chance of caring regularly for themselves.

This study depicted that students who obtained information from YouTube were more likely to experience prevention behaviors [AOR (CI) = 1.87 (1.19–2.91)]. This result was supported by a former study finding from Jordan, in which the use of social and news media platforms such as YouTube, Facebook, WhatsApp, and others, positively influence healthy behavioral changes and COVID-19 prevention (40). Besides, in the Amhara region, social media was a significant predictor of good knowledge and practice of COVID-19 (41). This could be because of quick access to up-to-date information and the availability of the service everywhere and every time using mobile data. The potential advantages of social media platforms include enhanced public awareness, improved health outcomes, promotion of healthy behavior, dissemination of public health interventions, and delivery of health news/facts to the community. However, informants, i.e., the World Health Organization, Government, and Ministry of Health (MOH), should assess the choice of media of students and the society to provide news/facts regarding COVID-19.

Students who had a high perception of the national efforts at controlling and preventing COVID-19 were three times more likely to have good prevention practices [AOR (CI)= 2.94 (2.04–4.25)]. A consistent finding was reported from the US, in which students with greater perceptions were more likely to implement health-protective behaviors to avoid COVID-19 (36). High perception is important for enhancing protection and control priorities against COVID-19 infection.

Because of the emergent nature of the COVID-19 pandemic, social immunization programs are required to maximize the health of the population. It is an essential program for attaining both the national and international goals in the control of the COVID-19 pandemic. To build trust, this needs to be conducted ethically with detailed counseling and education of health care providers and the community (42). However, the current vaccination coverage is insufficient to achieve pandemic control. Vaccine hesitancy become a growing challenge for social immunization programs. Vaccine hesitancy is a delay in acceptance or refusal of safe vaccines for themselves or their children despite the availability of vaccination services. Such perceptions might be the result of misinformation from mass media, political issues, the health system, and/or the community. Strengthening health literacy and counseling at all levels is required to improve COVID-19 vaccination uptake and reach (43). Vaccinated people had less risk of individual symptoms, severe disease, hospital admission, and death (44). The combination of vaccination and wearing a mask is potentially synergistic and is of high benefit in the prevention and control of COVID-19 (45).

### Limitations

Participants may have answered questions in a manner that would be viewed favorably by others and/or may not respond to the actual practice (social desirability bias). Other confounders like knowledge and attitude were not assessed in this study. Since this is cross-sectional, it fails to establish a causal relationship.

## Conclusion and Recommendation

During school reopening, most students had a low perception of national efforts at controlling COVID-19 and poor prevention practices. Socio-demographic background, having a chronic illness, information source, and perception of national efforts were factors of COVID-19 prevention practice. The findings will help instructors, health bureau managers, health care professionals, woreda, zonal, and university administrators, and other responsible bodies to organize the necessary interventional programs i.e., health education, law enforcement, award, and recognition for practitioners, to prevent further spread.

## Data Availability Statement

The raw data supporting the conclusions of this article will be made available by the authors, without undue reservation.

## Ethics Statement

The studies involving human participants were reviewed and approved by Debre Berhan University. The patients/participants provided their written informed consent to participate in this study.

## Author Contributions

MT conceived and designed the proposal, performed analysis, and prepared the manuscript. ST, GA, and GM critically revised, provided necessary comments, and made basic adjustments to the final article. All authors contributed to the article and approved the submitted version.

## Conflict of Interest

The authors declare that the research was conducted in the absence of any commercial or financial relationships that could be construed as a potential conflict of interest.

## Publisher's Note

All claims expressed in this article are solely those of the authors and do not necessarily represent those of their affiliated organizations, or those of the publisher, the editors and the reviewers. Any product that may be evaluated in this article, or claim that may be made by its manufacturer, is not guaranteed or endorsed by the publisher.
